# Community-Based HPV Self-Sampling to Enhance Access to Cervical Cancer Prevention: A Continuum-of-Care Model from Urban Nepal

**DOI:** 10.3390/curroncol33090539

**Published:** 2026-09-05

**Authors:** Shreekrishna Maharjan, Anamika Maharjan, Kushala K. Bista, Pranali G. Patel, Jitendra Pariyar, Pema Lhaki, Sadeep Shrestha

**Affiliations:** 1 Department of Biochemistry, Patan Academy of Health Sciences (PAHS), Lalitpur 44700, Nepal; shreekrishnamaharjan@pahs.edu.np; 2 Nepal Fertility Care Center (NFCC), Gusingal, Sanepa, Lalitpur 44600, Nepal; anamika@nfcc.org.np (A.M.); kushala@nfcc.org.np (K.K.B.); pema@nfcc.org.np (P.L.); 3 Department of Epidemiology, University of Alabama at Birmingham, Birmingham, AL 35294, USA; ppatel49@uab.edu; 4 Cancer Care Nepal and National Cancer Hospital, Lalitpur 44600, Nepal; jipariyar@yahoo.com

**Keywords:** human papillomavirus, cervical cancer, self-sampling, genotyping, community health screening, HPV DNA testing

## Abstract

Cervical cancer remains the leading cause of cancer-related mortality among women in Nepal, where national screening coverage is approximately 16%. This study evaluated the feasibility of a community-based, door-to-door self-sampling strategy for high-risk human papillomavirus (hrHPV) detection in an urban municipality of central Nepal and assessed hrHPV prevalence, genotype distribution, screening outcomes, and associated demographic factors. A cross-sectional study was conducted between September 2023 and May 2024 in Ward No. 3 of Lalitpur Metropolitan City. Women aged 30–60 years were recruited through trained community health workers, provided education and home-based self-sampling kits, and completed a demographic questionnaire. Dry cervical swabs from 418 participants were tested for hrHPV. Women with positive results underwent visual inspection with acetic acid (VIA), followed by colposcopy, biopsy when indicated, and thermal ablation for confirmed precancerous lesions. Overall participation was 64.1%, and hrHPV prevalence was 10.3%. Non-16/18 hrHPV genotypes predominated (55.8%), followed by HPV16 (20.9%). Women aged > 50 years were more likely to be hrHPV- and VIA-positive (OR = 7.39, *p* = 0.02). Six pre-cancer cases were identified: five cervical intraepithelial neoplasia (CIN1) and one CIN3 case. Community-based hrHPV self-sampling was found to be feasible and achieved effective linkage to triage and treatment, supporting its consideration for strengthening cervical cancer screening in Nepal.

## Simple Summary

Cervical cancer remains a major health problem in Nepal, yet many women do not participate in screening because of barriers such as travel, limited time, privacy concerns, and household responsibilities. This study evaluated the feasibility and acceptability of a community-based, door-to-door screening approach in which trained community health workers visited homes, provided education, and supported women in collecting their own samples for human papillomavirus (HPV) testing. Among the 418 asymptomatic women screened, approximately 1 in 10 tested positive for high-risk HPV, with several HPV genotypes identified. Women who tested positive were successfully linked to follow-up evaluation, including visual inspection with acetic acid (VIA), diagnostic assessment, and treatment when needed. The program was delivered through a partnership involving local government, community health workers, and private healthcare providers, creating a screen–triage–treat continuum. This community-centered model demonstrates high acceptability and feasibility and offers a scalable framework for expanding cervical cancer prevention services in Nepal.

## 1. Introduction

Cervical cancer, a preventable sequalae of persistent high-risk human papillomavirus (hrHPV) infection, remains a major global public health challenge. In 2024, an estimated 604,000 women were diagnosed with cervical cancer, and approximately 280,000 died from the disease worldwide, with over 94% of deaths occurring in low- and middle-income countries (LMICs) [1,2]. In Nepal, approximately 11.5 million women aged ≥15 years are at risk of developing cervical cancer [3]. Current conservative estimates indicate that about 1532 women are diagnosed with cervical cancer annually and 879 die from the disease [4]. Nepal’s age standardized incidence and mortality rates are 10.3 and 6.0 per 100,000 women, respectively [4].

Effective screening programs are essential for prevention; however, access remains limited in Nepal, where fewer than half of women aged 25–65 have ever undergone a pelvic examination and only 9–16% have ever been screened for cervical cancer [3,5,6]. Current national cervical cancer prevention strategies primarily focus on secondary prevention via facility-based opportunistic screening and periodic community health camps using visual inspection with acetic acid (VIA), Pap smears, and to a lesser extent, molecular HPV DNA testing targeted at women aged 30–60 years [7,8,9,10,11]. At the primary prevention level, Nepal has recently prioritized the expansion of HPV vaccination programs targeting adolescent girls [12]. However, uptake of both screening and preventive services remains suboptimal due to health infrastructure limitations, resource availability, and social barriers [13,14].

Persistent hrHPV infection is a necessary precursor to pre-cancerous lesions such as low-grade squamous intraepithelial lesions (LSIL) or high-grade squamous intraepithelial lesions (HSIL), and invasive cervical cancer too. Molecular hrHPV testing is now recommended by the World Health Organization (WHO) as a primary screening method, alone or combined with cytology or visual inspection with acetic acid (VIA), particularly in resource-constrained settings [3,9,15,16,17]. WHO guidelines further emphasize primary HPV testing through context-adapted implementation models that enhance uptake and retention across the full screen–triage–treat continuum [18,19].

Despite these advances, screening uptake in LMICs remains low due to barriers such as embarrassment, privacy concerns, stigma, limited awareness, reliance on spousal permission, and discomfort with pelvic examinations, disproportionately affecting home-bound women with limited autonomy [20]. Community-based, door-to-door outreach may be especially relevant in Nepal, where household responsibilities, female mobility constraints, privacy concerns, and the sensitivity of discussing gynecologic health can influence access to clinic-based screening. Previous work in Nepal has demonstrated the feasibility of HPV self-sampling in community settings [9,21,22]. Self-collected vaginal sampling for HPV testing addresses many of these barriers. Globally validated as comparable in sensitivity to clinician-collected samples, self-sampling improves privacy, comfort, and access by enabling home-based participation, allowing women to complete screening within the privacy and time flexibility of their own households [9,23,24,25].

The present study extends prior local feasibility evidence to a dense urban ward by evaluating a community-anchored model linking household outreach and HPV self-sampling with local testing and follow-up. Specifically, this study evaluates the feasibility, prevalence, and clinical outcomes of a community-based HPV self-sampling cervical cancer screening program in an urban local ward of central Nepal. The primary objectives of this study were to: (1) assess the feasibility and acceptability of a community-based, door-to-door, home-visit-driven HPV self-sampling model; (2) determine the prevalence and genotype distribution of hrHPV; (3) identify demographic and behavioral risk factors associated with hrHPV-positive women, and (4) critically analyze the performance of the subsequent screen–triage–treat care cascade, specifically identifying the proportion of loss to follow-up (LTFU). The findings from this study will help inform and strengthen strategies for scaling up the national cervical cancer screening program in Nepal.

## 2. Materials and Methods

### 2.1. Study Design and Population

This community-based analytical cross-sectional study was conducted as part of a cervical cancer screening program in Ward No. 3 of Lalitpur Metropolitan City, an urban area of Lalitpur District in central Nepal, between September 2023 and May 2024. The program followed a screen–triage–treat strategy consisting of (1) hrHPV testing using self-collected samples and subsequent genotyping for confirmation and distribution; (2) triage of hrHPV-positive women with VIA, colposcopy, and biopsy; and (3) treatment of pre-cancerous lesions with thermal ablation. Screening activities were coordinated through the ward local government office in partnership with a non-government organization (NGO), Nepal Fertility Care Center (NFCC) and a government community health clinic. All field personnel received centralized training in community engagement and study procedures.

To support community engagement and program delivery, a household survey was conducted in Ward 3 to identify women aged 30 to 60 years and collect household and contact information. These data were entered into a cluster-based database with unique identifiers corresponding to six ward clusters (Pulchowk, Dhobighat, Sihiti, Shantibasti, Jhamsikhel-1, and Jhamsikhel-2). Ward 3 Female Community Health Volunteers (FCHVs) and Tole Health Promoters (THPs) received two orientation sessions, initially on HPV infection and cervical cancer prevention, and subsequently on household outreach, participant education, completion of program documentation, and management of common implementation challenges. Screening information was disseminated through community groups, pamphlets, and Ward 3 social media channels. Trained FCHVs and THPs then conducted home visits, using an NFCC-developed Nepali-language flipchart to provide information on HPV, cervical cancer, risk factors, screening options, self-sampling procedures, possible results, referral pathways, and HPV vaccination. Following written informed consent, participants received self-sampling kits and collected samples at home. Samples were returned through community health workers and transported for HPV DNA testing at the Ward 3 community clinic. Women with negative results received counseling through the community network, whereas those testing positive for hrHPV were actively contacted, counseled, and linked to diagnostic evaluation and treatment. Follow-up across the screen–triage–treat continuum was jointly monitored by NFCC and Ward 3 stakeholders.

The target population included eligible women aged ≥30 years residing in the ward. Although the program targeted women aged 30–60 years, older women aged >60 years who expressed interest were also included. Eligible women were identified through the municipal residence registry and approached by trained Female Community Health Volunteers (FCHVs) and Tole Health Promoters (THPs). Inclusion criteria were willingness to undergo cervical cancer screening using self-sampling, ability to provide written informed consent, not currently menstruating, having an intact cervix, and not being pregnant. Women were excluded if they self-reported undergoing total hysterectomy, lacked a uterus, were receiving treatment for cervical cancer, were pregnant at the time of recruitment, or declined screening or self-sampling. Written informed consent was obtained from all participants prior to enrollment.

**Figure 1:**
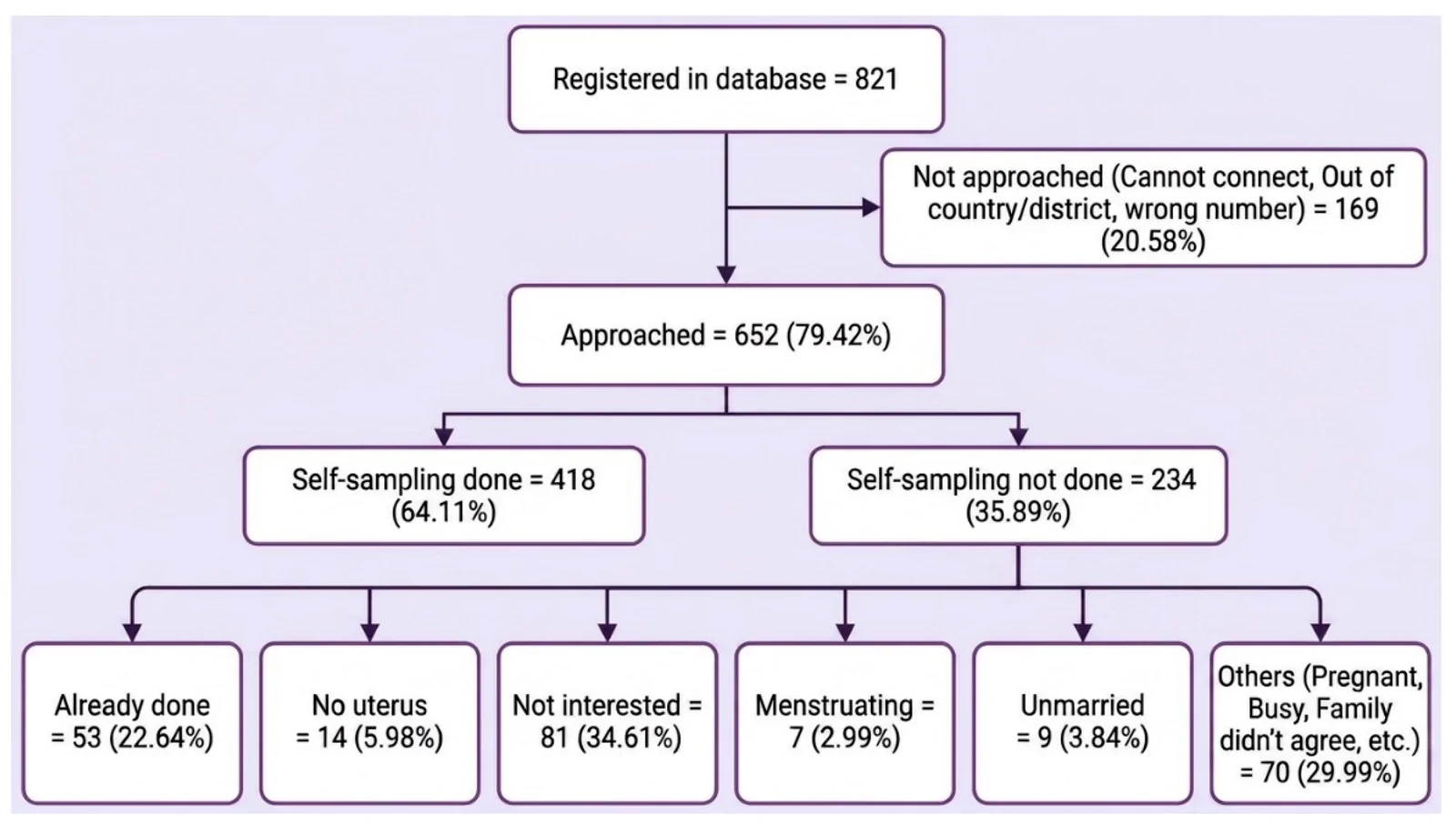
Participant recruitment and enrollment flow diagram. Identification, recruitment, enrollment, and completion of hrHPV self-sampling, with reasons for non-participation.

Of the 821 eligible registered women residing in the ward, 652 (79.42%) were successfully approached and 418 (64.11%) consented to self-sampling: participant flow, including reasons for non-approach and non-participation are presented in Figure 1.

### 2.2. Data Collection

Participants completed a structured questionnaire related to demographic characteristics (age, education, marital status, nationality, parity, age at marriage, number of pregnancies), socioeconomic factors (household income, occupation, migration work history), reproductive behaviors (lifetime number of sexual partners, sexual activity, age at first sexual intercourse, age at first menstruation, contraceptive use), risk factors (migration status, alcohol consumption, tobacco and cigarette use), and knowledge of sexually transmitted diseases (STDs), cervical cancer, HPV, and HPV vaccination. The questionnaire was administered by trained Nepali-speaking FCHVs and THPs. Confidentiality and anonymity were maintained throughout data collection.

### 2.3. Sampling, HPV Screening, and Genotyping

FCHVs and THPs conducted home visits to provide culturally appropriate education on HPV and cervical cancer in local language. After informed consent was given, women received a self-sampling kit containing written instructions and a sterile dry brush swab. Participants self-collected vaginal samples and returned them within 24 h, and samples were labeled and transported to the Ward No. 3 community clinic, where they were stored at −80 °C.

HPV DNA testing was performed using the validated AmpFire HPV Screening 16/18/HR assay (ATILA BioSystems, Sunnyvale, CA, USA) according to the manufacturer’s protocols, detecting 14 high-risk HPV types (oncogenic types 16, 18, 31, 33, 35, 39, 45, 51, 52, 56, 58, 59, 66, and 68) [26]. The initial screening assay reported HPV16 or HPV18 and a pooled hrHPV positivity for the rest of the 12 high-risk types. All samples positive for the pooled hrHPV underwent genotyping using the AmpFire High-Risk Genotyping assay (ATILIA BioSystems, Sunnyvale, CA, USA) to identify individual genotypes. Each assay batch included positive and negative quality controls and internal controls to ensure assay validity and sample adequacy.

### 2.4. Screen–Triage–Treat Cascade and Follow Up

**Figure 2:**
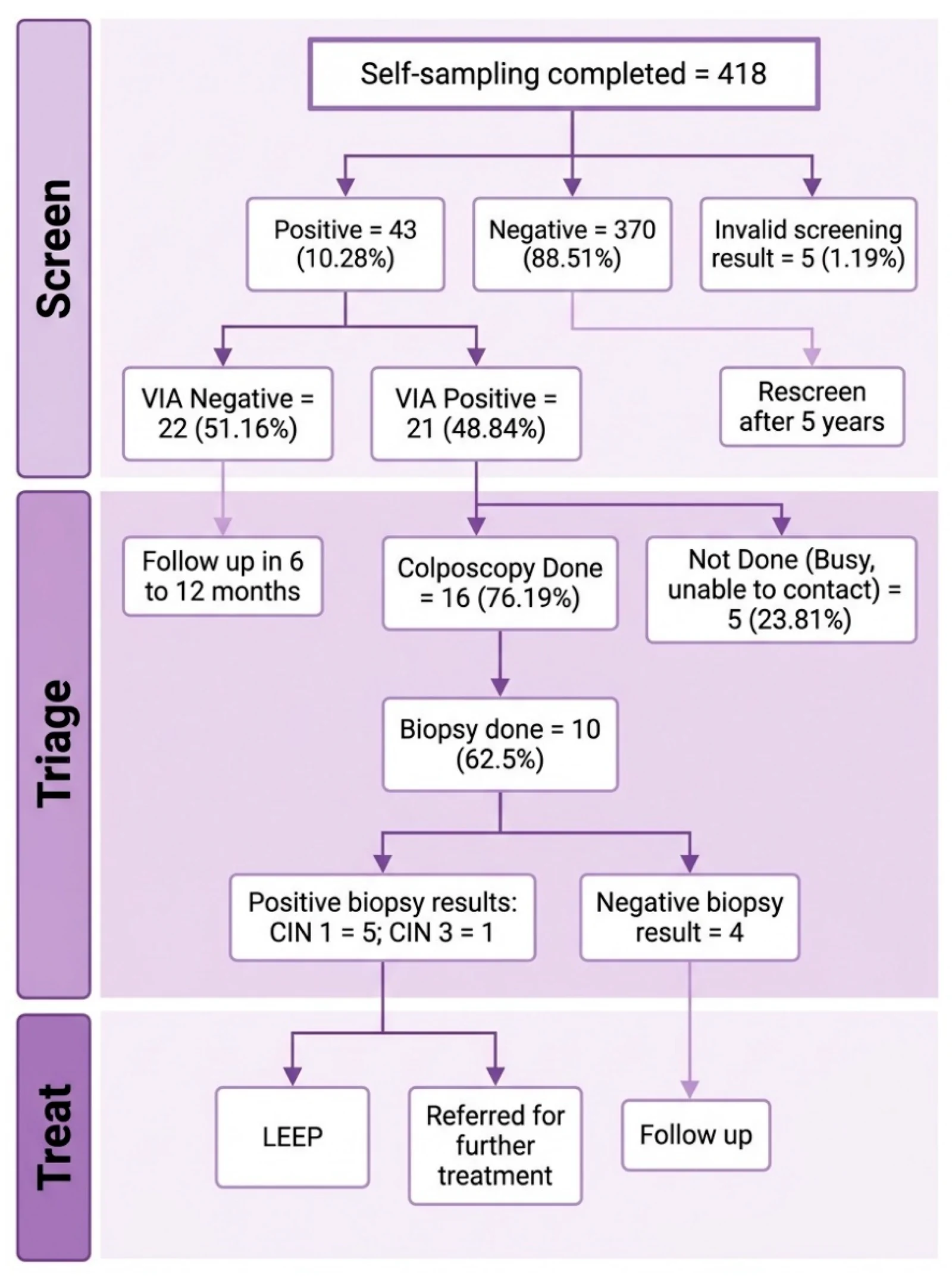
Screening–triage–treat cascade for cervical cancer prevention. hrHPV screening outcomes, VIA triage, colposcopy and biopsy findings, and treatment or follow-up of screen-positive women.

A standardized patient report was generated and verified by a registered laboratory professional. Women with hrHPV-negative results were informed and counseled to rescreen in five years, in accordance with national guidelines [27,28]. Women with hrHPV-positive results were contacted immediately, consulted and referred to a gyneco-oncology clinician for visual inspection with VIA triage. Women with VIA-negative findings were advised to return for follow-up in 6–12 months, while VIA-positive women were scheduled for colposcopy and biopsy when indicated. Biopsies of acetowhite lesions were evaluated histopathologically to confirm CIN grade at the National Cancer Hospital in Lalitpur. Women diagnosed with CIN requiring treatment were referred for oncologic management. Follow-up adherence and outcomes were tracked throughout the care cascade, with active outreach for missed appointments (Figure 2).

### 2.5. Data Analysis

Descriptive statistics were used to summarize demographic categorical variables and to report screening uptake, hrHPV prevalence and genotype frequencies. Means, standard deviations (SD), medians, inter-quartile ranges (IQR), and percentages (%) were calculated as appropriate. Bivariate analyses were performed using χ^2^ test or Fisher’s exact test for categorical variables and the Mann–Whitney U test for continuous variables to evaluate for associations. Univariable logistic regression models were used to estimate odds ratios (ORs) and 95% confidence intervals (CIs) for factors associated with hrHPV positivity and hrHPV/VIA positivity. Because of the limited number of outcome events, particularly within the hrHPV/VIA-positive subgroup, multivariable analyses were not performed to avoid model overfitting and unstable estimates.

## 3. Results

### 3.1. Participant Flow

Overall participation (Table 1) and reasons for non-participation are presented in Figure 1.

### 3.2. Screen, Triage, and Treatment Flow

Of the 418 self-collected samples, 10.28% (n = 43) samples tested positive for hrHPV (Table 2). Among the hrHPV-positive cases, HPV16 and HPV18 single infections accounted for 25.58% (n = 11) and 4.65% (n = 2), respectively, while 55.81% (n = 24) were positive for other hrHPV types. Co-infections included HPV16/HPV18 (2.33%, n = 1), HPV16 with other hrHPV types (9.30%, n = 4), and HPV18 with other hrHPV types (2.33%, n = 1). The follow-up pathway for the 43 hrHPV-positive women is detailed in Figure 2.

The screen–triage cascade, including VIA triage, colposcopy, and biopsy completion, is presented in Figure 2. Histopathology confirmed cervical intraepithelial neoplasia (CIN) in six women, CIN1 in five cases, and CIN3 in one case (Table 3). All CIN1 cases were treated with thermal ablation and scheduled for surveillance, while the CIN3 case was referred to a tertiary center. Five women did not perform VIA for reasons such as busy schedules and/or loss to follow-up.

### 3.3. Patient Characteristics

No significant demographic differences were observed between hrHPV-positive and hrHPV-negative groups. The median age of the participants was 48 years, ranging from 30 to 63 years: the majority of the women in the study were ≥50 years (n = 160; 38.8%), followed by those aged 40–49 years (n = 158; 38.3%), and lastly those aged 30–39 years (n = 95; 23.0%). Most participants had a secondary education (n = 141, 34.3%), followed by a post-secondary education (n = 117; 28.2%), were married (n = 389; 94.2%), and reported sexual activity (n = 396; 96.6%). The median age at first sexual intercourse was 21.5 years, and the median age at menarche was 14 years. Nearly all women self-reported having one lifetime sexual partner (n = 313; 97.2%), and most had been pregnant at least once (n = 381; 95.7%). The predominant occupation was office work (n = 234; 58.8%), followed by business (n = 57; 17.3%). Alcohol consumption was common (n = 242; 59.7%), while tobacco (n = 7; 2.2%) and cigarette use (n = 13; 3.4%) was low overall. Nearly half of the participants (n = 177; 46.3%) reported husbands with migrant work experience. Awareness of cervical cancer was high (n = 377; 92.4%), but fewer had heard of HPV (n = 185 45.6%) or the HPV vaccine (n = 189; 46.2%). Most women (n = 386; 94.4%) indicated willingness to vaccinate their child if the vaccine was free (Table 1).

### 3.4. Characteristics of HPV/VIA Positive Women

Among the HPV-positive women (n = 43; 10.4%), the highest proportion was observed in the 40–49 years age group (n = 19; 44.2%), followed by the 50+ years age group (n = 16; 37.2%) and then the 30–39 years age group (n = 8; 18.6%). Univariate analysis showed that age at first sexual intercourse (*p* = 0.004) was significantly associated with hrHPV positivity, while marital status (*p* = 0.08) and whether the participant had ever had sexual intercourse (*p* = 0.07) showed non-statistically significant trends (Table 3). HPV infection was marginally higher, although not statistically significant, among single women who were either divorced or widowed compared to married women (n = 5; 11.6% vs. n = 24; 5.8%, *p* = 0.09). HPV positivity did not differ significantly by history of multiple marriages, sexual activity, or parity.

A significant association was observed between the over 50 age group and HPV/VIA positivity (χ^2^ test, *p* = 0.02). The median age was slightly higher among HPV^+^/VIA^+^ women (48 years) compared with HPV-negative women (46 years). Within the HPV^+^/VIA^+^ subgroup (n = 21; 48.8%), the majority of the women were in the 50+ age group (n = 12; 57.1%). Conversely, most HPV^+^/VIA^−^ cases (n = 22; 51.2%) occurred in women in the 40–49 years age group (n = 12; 54.5%). There was a trending association between educational level and HPV^+^/VIA^+^ (*p* = 0.06).

Given the small number of hrHPV-positive (n = 43) and hrHPV/VIA-positive (n = 21) cases, we restricted our analyses to univariable models and considered the effect estimates to be exploratory.

## 4. Discussion

This community-based study conducted in Lalitpur, Nepal, demonstrates that a door-to-door cervical cancer screening strategy using culturally tailored HPV self-sampling, implemented through collaboration between local government and a non-government social service organization, is both feasible and effective in a dense urban setting. High community participation, detection of clinically meaningful hrHPV and precancer prevalence, and strong adherence across the follow-up cascade indicate that this model successfully mitigates key structural and sociocultural barriers to screening access. Collectively, these findings support the potential of this integrated, community-anchored approach as a scalable framework for strengthening cervical cancer screening programs in Nepal and similar resource-constrained urban contexts.

The overall hrHPV prevalence of 10.28% is consistent with prior estimates from Nepal [29] and comparable age-matched populations globally [30]. The predominance of non-16/18 hrHPV genotypes (55.81%) mirrors patterns observed in South Asia [31,32]. Earlier age at sexual debut was inversely associated with hrHPV positivity (OR = 0.88; *p* = 0.004), reflecting increased cumulative exposure, as previously documented [33,34]. The elevated risk among migrants (OR = 3.00; *p* = 0.06) indicates the interplay of complex social and behavioral factors, such as mobility, higher number of sex partners, and different access to health information, that may influence infection patterns. Higher hrHPV and VIA positivity in women > 50 raises questions about reactivation versus new infection [35,36], highlighting the need to assess the value of screening older women.

hrHPV positivity trended higher, though not statistically significantly, among previously married women (Table 1), though marital status alone may not independently predict infection in settings with low partner variation and predominantly monogamous relationships. Heterogeneity in sexual history among married participants, along with possible immune or hormonal changes following marital transitions, may partly explain this association, which aligns with evidence linking marital instability to increased HPV risk. VIA positivity was also significantly associated with older age (median 48.5 years, *p* = 0.04) and higher parity (≥2), consistent with biological pathways involving cumulative exposure and cervical trauma leading to persistent infection and precancer. Together, these findings support universal screening followed by community-level risk stratification of triage and treatment resources, prioritizing older, multiparous women for timely follow-up.

While self-sampling enabled successful enrollments, the care cascade revealed high engagement alongside ongoing systemic challenges. Notably, 81.4% of hrHPV-positive women completed facility-based VIA triage, higher than retention rates typically reported in multi-step screening programs across LMICs, suggesting that post-test counseling effectively motivates follow-up [15]. The detection and treatment of precancerous lesions, including one CIN3, highlight the model’s clinical impact. This community-based screen–triage–treat approach is especially valuable in resource-limited settings, where multiple visits often result in loss to follow-up.

This program demonstrates the feasibility of integrating HPV self-sampling within a continuum-of-care model that links screening, diagnostic evaluation, and treatment services in an urban Nepalese setting. Leveraging existing community health networks facilitated outreach and participation, while coordinated referral pathways supported timely follow-up and management of screen-positive women. The high completion of follow-up screening observed in this program suggests that integrating services across the cervical cancer care pathway may help reduce barriers to prevention and improve continuity of care. These findings provide valuable evidence for the implementation of HPV-based screening programs and may inform efforts to strengthen cervical cancer prevention services in other resource-constrained settings.

This study is limited by its cross-sectional design, which precludes assessment of temporality and long-term outcomes. Despite high screening uptake, attrition occurred during follow-up, with 18.6% of hrHPV-positive women not completing VIA and 23.8% of VIA-positive women not undergoing biopsy, highlighting opportunities to strengthen patient navigation. In addition, the small number of hrHPV-positive (n = 43) and hrHPV/VIA-positive (n = 21) cases restricted analyses to univariable models and resulted in wide confidence intervals. Consequently, observed associations, including the higher odds of hrHPV/VIA positivity among women aged >50 years, should be interpreted cautiously and validated in larger studies.

## 5. Conclusions

Overall, this community-based HPV self-sampling program provides a practical model for advancing equitable cervical cancer prevention in resource-constrained settings. Through collaboration between local government, community health workers, and referral services, the program achieved substantial screening uptake, effective linkage to care, and detection of clinically significant precancerous lesions. As countries work toward WHO cervical cancer elimination targets, integrating community-centered HPV screening and streamlined screen–triage–treat pathways may offer an effective strategy to reduce access disparities and strengthen population-level cervical cancer prevention in Nepal and similar LMIC settings.

## Figures and Tables

**Table 1:** Demographic and social characteristics of study participants.

Demographics	Total N (%)	HPV^+^ N (%)	HPV^+^/VIA^+^ N (%)	HPV^+^/VIA^−^ N (%)	HPV^−^ N (%)
**Age (years)**	**413**	**43**	**21**	**22**	**370**
30–39	95 (23.0)	8 (18.6)	2 (9.5)	6 (27.3)	87 (23.5)
40–49	158 (38.3)	19 (44.2)	7 (33.3)	12 (54.5)	139 (37.6)
50+	160 (38.8)	16 (37.2)	12 (57.1)	4 (18.2)	144 (38.9)
**Median (IQR)**	46 (40–53)	48 (40–53)	52 (45–56)	43 (38–48)	46 (40–53)
**Educational status**	**411**	**43**	**21**	**22**	**368**
No education	36 (8.8)	6 (14.0)	9 (42.9)	4 (18.2)	30 (8.1)
Primary education	118 (28.7)	12 (27.9)	4 (19.0)	1 (4.5)	106 (28.8)
Secondary education	141 (34.3)	13 (30.2)	4 (19.0)	9 (40.9)	128 (34.8)
Post-Secondary education	116 (28.2)	12 (27.9)	4 (19.0)	8 (36.4)	104 (28.3)
**Marital status**	**413**	**43**	**21**	**22**	**370**
Married	389 (94.2)	38 (88.4)	19 (90.5)	19 (86.4)	351 (94.9)
Others (widowed, divorced)	24 (5.8)	5 (11.6)	2 (9.5)	3 (13.6)	19 (5.1)
**Multiple marriage**	**405**	**39**	**21**	**18**	**366**
Yes	36 (8.9)	3 (7.7)	2 (9.5)	1 (5.6)	33 (9.0)
No	369 (91.1)	36 (92.3)	19 (90.5)	17 (94.4)	333 (91.0)
**Ever had sexual intercourse**	**410**	**41**	**20**	**21**	**369**
Yes	396 (96.6)	38 (92.7)	20 (100.0)	18 (85.72)	358 (97.02)
No	5 (1.22)	0 (0.0)	0 (0.0)	0 (0.00)	5 (1.35)
Did not want to say	9 (1.46)	3 (7.3)	0 (0.0)	3 (14.28)	6 (1.63)
**Age at first sexual intercourse, median (IQR)**	21 (19–25)	20 (17–23)	18 (17–21)	22 (18–24)	22 (19–25)
**Age at first menstruation, median (IQR)**	14 (12–16)	14 (12–16)	14 (12–16)	14 (11–17)	14 (12–16)
**Number of lifetime sexual partners**	**322**	**32**	**16**	**16**	**290**
1	313 (97.2)	32 (100.0)	16 (100.0)	16 (100.0)	281 (96.9)
2+	9 (2.8)	0 (0.0)	0 (0.0)	0 (0.0)	9 (3.1)
**Number of pregnancies**	**398**	**38**	**20**	**18**	**360**
0	17 (4.3)	0 (0.0)	0 (0.0)	0 (0.0)	17 (4.7)
1	106 (26.6)	10 (26.3)	4 (20.0)	6 (33.33)	96 (26.7)
2+	275 (69.1)	28 (73.7)	16 (80.0)	12 (66.67)	247 (68.6)
**Monthly income of family (Nepali Rs)**	**201**	**22**	**8**	**14**	**179**
<19,550	22 (10.9)	1 (4.6)	0 (0.0)	1 (7.1)	21 (11.7)
19,550 to 50,000	105 (52.2)	18 (81.8)	8 (100.0)	10 (71.5)	87 (48.7)
50,000 to 100,000	58 (28.9)	3 (13.6)	0 (0.00)	3 (21.4)	55 (30.7)
>100,000	16 (8.0)	0 (0.0)	0 (0.0)	0 (0.0)	16 (8.9)
**Occupation**	**398**	**42**	**20**	**22**	**356**
Business	57 (14.3)	3 (7.1)	0 (0.0)	3 (13.6)	54 (15.2)
Office worker	234 (58.8)	29 (69.1)	15 (75.0)	14 (63.7)	205 (57.6)
Housewife	29 (7.3)	1 (2.4)	1 (5.0)	0 (0.0)	28 (7.9)
Others	78 (19.6)	9 (21.4)	4 (20.0)	5 (22.7)	69 (19.4)
**Migrant work experience (husband)**	**382**	**35**	**16**	**19**	**347**
Yes	177 (46.3)	20 (57.1)	8 (50.0)	12 (63.2)	157 (45.2)
No	205 (53.7)	15 (42.9)	8 (50.0)	7 (36.8)	190 (54.8)
**Alcohol use**	**406**	**42**	**20**	**22**	**364**
Yes	242 (59.6)	23 (54.8)	9 (45.0)	14 (63.6)	219 (60.2)
No	164 (40.4)	19 (45.2)	11 (55.0)	8 (36.4)	145 (39.8)
**Tobacco product use**	**402**	**41**	**20**	**21**	**361**
Yes/Sometimes	7 (1.7)	1 (2.4)	1 (5.0)	0 (0.0)	6 (1.7)
Do not use	395 (98.3)	40 (97.6)	19 (95.0)	21 (100.0)	355 (98.3)
**Cigarette use**	**405**	**41**	**20**	**21**	**364**
Yes/Sometimes	13 (3.2)	2 (4.9)	1 (5.0)	1 (4.8)	11 (3.0)
Do not use	392 (96.8)	39 (95.1)	19 (95.0)	20 (95.2)	353 (97.0)
**Have you heard of STDs?**	**412**	**42**	**20**	**22**	**370**
Yes	346 (84.0)	33 (78.6)	12 (60.0)	21 (95.5)	313 (84.6)
No	66 (16.0)	9 (21.4)	8 (40.0)	1 (4.5)	57 (15.4)
**Have you heard of cervical cancer?**	**408**	**42**	**20**	**22**	**366**
Yes	377 (92.4)	40 (95.2)	18 (90.0)	22 (100.0)	337 (92.1)
No	31 (7.6)	2 (4.8)	2 (10.0)	0 (00.00)	29 (7.9)
**Have you heard of HPV?**	**406**	**42**	**20**	**22**	**364**
Yes	185 (45.6)	15 (35.7)	8 (40.0)	7 (31.8)	170 (46.7)
No	221 (54.4)	27 (64.3)	12 (60.0)	15 (68.2)	194 (53.3)
**Do you know of the HPV vaccine?**	**409**	**43**	**21**	**22**	**366**
Yes	189 (46.2)	14 (32.6)	5 (23.8)	9 (40.9)	175 (47.8)
No	220 (53.8)	29 (67.4)	16 (76.2)	13 (59.1)	191 (52.2)
**If free, would you give the HPV vaccine to your child?**	**409**	**43**	**21**	**22**	**366**
Yes	386 (94.4)	39 (90.7)	19 (90.5)	20 (91.0)	347 (94.8)
Do not know/did not want to say	15 (3.7)	3 (7.0)	2 (9.5)	1 (4.5)	12 (3.3)
Ask family	8 (2.0)	1 (2.3)	0 (0.0)	1 (4.5)	7 (1.9)

Data in bold shows the total individuals with complete data for the variable.

**Table 2:** Participants’ test results for HPV DNA screening of self-sample dry cervical swabs.

Category	Frequency (*f*)	Percentage (%)
HPV-Positive (16, 18 and * others)	43	10.28
− HPV16 only	11	25.58
− HPV18 only	2	4.65
− HPV (others)	24	55.81
− HPV16 and 18	1	2.33
− HPV16 and others	4	9.30
− HPV18 and others	1	2.33
− HPV16, 18 and others	0	0.00
**HPV-Negative**	370	88.52
**Invalid Results**	5	1.2%
**Total Samples Screened for HPV**	418	100.0

* Other oncogenic types (31, 33, 35, 39, 45, 51, 52, 56, 58, 59, 66 and 68).

**Table 3:** Univariate analysis of risk factors for HPV infection and VIA positivity.

Demographic Variables	hrHPV Positivity		hrHPV/VIA Positivity	
	Univariate		Univariate	
	OR (95% CI)	*p*-Value	OR (95% CI)	*p*-Value
**Age (years)**				
30–39	1		1	
40–49	1.44 (0.63–3.53)	0.392	1.55 (0.3–9.97)	0.61
50+	1.18 (0.50–2.93)	0.714	7.39 (1.3–54.6)	**0.02**
**Educational status**				
No education	1		1	
Primary education	0.88 (0.28–2.41)	0.804	1.42 (0.18–18.17)	0.75
Secondary education	0.73 (0.32–1.63)	0.437	0.22 (0.04–1.04)	0.06
Post-Secondary education	0.83 (0.36–1.89)	0.652	0.25 (0.045–1.19)	0.08
**Marital status**				
Married	1		1	
Others (widowed, divorced, and single)	2.58 (0.86–6.64)	0.087	0.71 (0.11–4.06)	0.7
**Multiple marriage**				
No	1		1	
Yes	0.97 (0.25–2.74)	0.962	1.49 (0.18–18.17)	0.71
**Age at first sexual intercourse**	0.88 (0.80–0.96)	**0.004**	0.89 (0.74–1.03)	0.12
**Age at first menstruation**	1.15 (0.97–1.36)	0.113	1.07 (0.76–1.5)	0.69
**Number of lifetime sexual partners**				
1	1			
2+	0.46 (0.00–3.74)	0.546		
**Number of pregnancies**				
2+	1		1	
1	0.25 (0.00–1.92)	0.229		
0	0.94 (0.43–1.94)	0.881	0.52 (0.12–2.12)	0.37
**Monthly income of family (Nepali Rs)**				
<19,550	1		1	
19,550 to <50,000	2.72 (0.63–27.11)	0.199	2.14 (0.1–330.3)	0.64
50,000 to 100,000	0.87 (0.14–9.03)	0.888	1.8 (0.05–330.3)	0.76
>100,000	0.66 (0.05–9.03)	0.729	1 (0.003–270.43)	1
**Occupation**				
Office worker	1		1	
Business	0.45 (0.12–1.25)	0.134	0.14 (0.001–1.55)	0.12
Housewife	0.37 (0.04–1.49)	0.182	2.72 (0.14–403.43)	0.51
Others	0.95 (0.42–2.01)	0.902	0.76 (0.17–3.32)	0.72
**Migrant work experience**				
No	1		1	
Out of district	2.80 (0.97–7.36)	0.056	1.58 (0.27–11.02)	0.62
Out of country	1.38 (0.64–2.93)	0.406	0.38 (0.08–1.6)	0.19
**Alcohol use**				
No	1	0.49	1	0.24
Yes	0.80 (0.42–1.52)		0.48 (0.14–1.6)	
**Tobacco product use**				
Do not use	1		1	
Yes/Sometimes	2.03 (0.21–9.98)	0.476	3.32 (0.17–492.75)	0.44
**Cigarette use**				
Do not use	1		1	
Yes/Sometimes	1.95 (0.37–6.92)	0.385	1.05 (0.08–13.46)	0.97
**Have you heard of STDs?**				
Yes	1	0.316	1	0.19
No	0.67 (0.3–1.47)		0.07 (0.01–0.64)	
**Have you heard of cervical cancer?**				
Yes	1	0.415	1	0.99
No	1.84 (0.42–7.98)		0.00 (0.00-Inf)	
**Have you heard of HPV?**		0.475		
Yes	1		1	0.58
No	0.78 (0.39–1.55)		1.43 (0.4–5.07)	
**Do you know of the HPV vaccine?**		0.061		
Yes	1		1	0.38
No	0.53 (0.27–1.03)		0.45 (0.12–1.68)	
**If free, would you give the HPV vaccine to your child?**				
Yes	1	0.275	1	
No	0.53 (0.17–1.65)		0.95 (0.12–7.44)	0.96

*p*-value < 0.05 are bolded.

## Data Availability

The datasets generated and analyzed during the current study are available from the corresponding author upon reasonable request, in accordance with institutional policy and participant confidentiality agreements. Deidentified summary data may be shared upon approval by the NHRC.

## Abbreviations

The following abbreviations are used in this manuscript:

CI	Confidence Interval
CIN	Cervical Intraepithelial Neoplasia
FCHV	Trained Female Community Health Volunteer
hrHPV	High-Risk Human Papillomavirus
HSIL	High-Grade Squamous Intraepithelial Lesions
IQR	Inter-Quartile Ranges
LMICs	Low- and Middle-Income Countries
LSIL	Low-grade Squamous Intraepithelial Lesions
LTFU	Loss To Follow-Up
NGO	Non-Government Organization
OR	Odds Ratio
SD	Standard Deviation
STD	Sexually Transmitted Disease
THP	Tole Health Promoters
VIA	Visual Inspection with Acetic acid
WHO	World Health Organization

## References

[1] World Health Organization Cervical Cancer. https://www.who.int/news-room/fact-sheets/detail/cervical-cancer.

[2] Sung H., Filho A.M., Laversanne M., Ferlay J., Siegel R.L., Soerjomataram I., Jemal A., Bray F. (2026). Global cancer statistics 2024: GLOBOCAN estimates of incidence and mortality worldwide for 34 cancers in 186 countries. CA Cancer J. Clin..

[3] Bruni L., Albero G.Z., Serrano B., Mena M., Collado J.J., Gómez D., Muñoz J., Bosch F.X., de Sanjosé S. (2023). ICO/IARC Information Centre on HPV and Cancer (HPV Information Centre). Human Papillomavirus and Related Diseases in Nepal.

[4] International Agency for Research on Cancer (IARC) (2026). Nepal Fact Sheet: GLOBOCAN 2024 Estimates of Incidence and Mortality. Global Cancer Observatory, World Health Organization. https://gco.iarc.who.int.

[5] Johnson D.C., Bhatta M.P., Gurung S., Aryal S., Lhaki P., Shrestha S. (2014). Knowledge and awareness of human papillomavirus (HPV), cervical cancer and HPV vaccine among women in two distinct Nepali communities. Asian Pac. J. Cancer Prev..

[6] Sankaranarayanan R., Bhatla N., Gravitt P.E., Basu P., Esmy P.O., Ashrafunnessa K., Ariyaratne Y., Shah A., Nene B.M. (2008). Human papillomavirus infection and cervical cancer prevention in India, Bangladesh, Sri Lanka and Nepal. Vaccine.

[7] Family Welfare Division, Department of Health Services, Ministry of Health and Population, Nepal (2024). Cervical Cancer Screening HPV DNA SOP 2080 [Internet].

[8] Bhatta M.P., Johnson D.C., Lama M., Aryal S., Lhaki P., Shrestha S. (2017). High-risk human papillomavirus infection and abnormal cervical cytology among Nepali and Bhutanese refugee women living in eastern Nepal. BMC Infect. Dis..

[9] Johnson D.C., Bhatta M.P., Smith J.S., Kempf M.C., Broker T.R., Vermund S.H., Chamot E., Aryal S., Lhaki P., Shrestha S. (2014). Assessment of high-risk human papillomavirus infections using clinician- and self-collected cervical sampling methods in rural women from far western Nepal. PLoS ONE.

[10] Shakya S., Syversen U., Asvold B.O., Bofin A.M., Aune G., Nordbo S.A., Vaidya K.M., Karmacharya B.M., Afset J.E., Tingulstad S. (2017). Prevalence of human papillomavirus infection among women in rural Nepal. Acta Obstet. Gynecol. Scand..

[11] Thapa N., Maharjan M., Shrestha G., Maharjan N., Petrini M.A., Zuo N., He C., Yang J., Xu M., Ge C. (2018). Prevalence and type-specific distribution of human papillomavirus infection among women in mid-western rural, Nepal- A population-based study. BMC Infect. Dis..

[12] World Health Organization (2025). Accelerating Cervical Cancer Elimination Through HPV Vaccination in Nepal [Internet].

[13] Shrestha A.D., Andersen J.G., Gyawali B., Shrestha A., Shrestha S., Neupane D., Ghimire S., Campbell C., Kallestrup P. (2022). Cervical cancer screening utilization, and associated factors, in Nepal: A systematic review and meta-analysis. Public Health.

[14] (2022). Overview on Cervical Cancer Screening Program in Nepal.

[15] (2010). Human Papillomavirus and Related Cancers in Nepal.

[16] WHO (2016). WHO Information Centre on HPV and Cervical Cancer, Human Papillomavirus and Related Cancers, Fact Sheet 2016.

[17] Munoz N., Bosch F.X., de Sanjose S., Herrero R., Castellsague X., Shah K.V., Snijders P.J., Meijer C.J., for the International Agency for Research on Cancer Multicenter Cervical Cancer Study Group (2003). Epidemiologic classification of human papillomavirus types associated with cervical cancer. N. Engl. J. Med..

[18] World Health Organization (2026). WHO Guideline for Screening and Treatment of Cervical Pre-Cancer Lesions for Cervical Cancer Prevention: Use of Human Papillomavirus (HPV) DNA Genotyping.

[19] World Health Organization (2026). Evidence-Informed Guidance for the Implementation of HPV-Based Cervical Cancer Screening Programmes.

[20] Petersen Z., Jaca A., Ginindza T.G., Maseko G., Takatshana S., Ndlovu P., Zondi N., Zungu N., Varghese C., Hunting G. (2022). Barriers to uptake of cervical cancer screening services in low-and-middle-income countries: A systematic review. BMC Womens Health.

[21] Dixit S., Maharjan A., Gutierrez M., Bista K.K., Patel P.G., Khanna S., Maharjan S., Pariyar J., Boitano T.L., Jolly P.E. (2026). Public Space Specimen Self-collection as an Additional Cervical Cancer Screening Approach in Urban Setting in Kathmandu, Nepal. Asian Pac. J. Cancer Prev..

[22] Shakya S., Paneru B., Uprety S., Acharya Y., Shrestha S., Karmacharya A., Makaju S., Spiegelman D., Sheth S.S., Shrestha A. (2025). Acceptability of Self-sampling in Human Papillomavirus Deoxyribonucleic acid based Cervical Screening in Nepal: A Mixed-Methods Study. Kathmandu Univ. Med. J. (KUMJ).

[23] Fitzpatrick M.B., Behrens C.M., Hibler K., Parsons C., Kaplan C., Orso R., Parker L., Memmel L., Collins A., McNicholas C. (2025). Clinical Validation of a Vaginal Cervical Cancer Screening Self-Collection Method for At-Home Use: A Nonrandomized Clinical Trial. JAMA Netw. Open.

[24] Gibert M.J., Sanchez-Contador C., Artigues G. (2023). Validity and acceptance of self vs conventional sampling for the analysis of human papillomavirus and Pap smear. Sci. Rep..

[25] Costa S., Verberckmoes B., Castle P.E., Arbyn M. (2023). Offering HPV self-sampling kits: An updated meta-analysis of the effectiveness of strategies to increase participation in cervical cancer screening. Br. J. Cancer.

[26] Hou J., Belinson J.L., Du H., Li C., Zhang W., Zhang L., Zhang Y., Qu X., Wu R. (2024). AmpFire HPV and ScreenFire RS HPV validation trial. Am. J. Clin. Pathol..

[27] Ministry of Health and Population (MoHP), Nepal (2010). National Guidelines for Cervical Cancer Screening and Prevention. Kathmandu: MoHP.

[28] Cervical Cancer Screening and Prevention (CCSP) in Nepal (2015). A Reference Manual 2015 Department of Health Services, Ministry of Health and Population.

[29] Paudel P., Sah A. (2025). A meta-analysis of the prevalence, genotype distribution and risk factors for human papillomavirus infection in Nepal. PLoS ONE.

[30] Sabeena S., Bhat P.V., Kamath V., Bhat S.K., Nair S., Chandrabharani K., Arunkumar G. (2017). Community-Based Prevalence of Genital Human Papilloma Virus (HPV) Infection: A Systematic Review and Meta-Analysis. Asian Pac. J. Cancer Prev..

[31] Han S., Lin M., Liu M., Wu S., Guo P., Guo J., Xie L., Qiu S., Xu A., Cai Y. (2025). Prevalence, trends, and geographic distribution of human papillomavirus infection in Chinese women: A summative analysis of 2,728,321 cases. BMC Med..

[32] Okoye J.O., Chukwukelu C.F., Okekpa S.I., Ogenyi S.I., Onyekachi-Umah I.N., Ngokere A.A. (2021). Racial Disparities Associated with the Prevalence of Vaccine and Non-Vaccine HPV Types and Multiple HPV Infections between Asia and Africa: A Systematic Review and Meta-Analysis. Asian Pac. J. Cancer Prev..

[33] Rositch A.F., Patel E.U., Petersen M.R., Quinn T.C., Gravitt P.E., Tobian A.A.R. (2021). Importance of Lifetime Sexual History on the Prevalence of Genital Human Papillomavirus (HPV) Among Unvaccinated Adults in the National Health and Nutrition Examination Surveys: Implications for Adult HPV Vaccination. Clin. Infect. Dis..

[34] Bosch F.X., Burchell A.N., Schiffman M., Giuliano A.R., de Sanjose S., Bruni L., Tortolero-Luna G., Kjaer S.K., Munoz N. (2008). Epidemiology and natural history of human papillomavirus infections and type-specific implications in cervical neoplasia. Vaccine.

[35] Gravitt P.E., Rositch A.F., Silver M.I., Marks M.A., Chang K., Burke A.E., Viscidi R.P. (2013). A cohort effect of the sexual revolution may be masking an increase in human papillomavirus detection at menopause in the United States. J. Infect. Dis..

[36] Osmani V., Horner L., Nkurunziza T., Rank S., Tanaka L.F., Klug S.J. (2025). Global prevalence of cervical human papillomavirus in women aged 50 years and older with normal cytology: A systematic review and meta-analysis. Lancet Microbe.

